# The Role of Executive Functions in Social Cognition among Children with Down Syndrome: Relationship Patterns

**DOI:** 10.3389/fpsyg.2016.01363

**Published:** 2016-09-13

**Authors:** Anna Amadó, Elisabet Serrat, Eduard Vallès-Majoral

**Affiliations:** ^1^Department of Psychology, University of GironaGirona, Spain; ^2^Servei Neuropsicopedagògic ArlotGirona, Spain

**Keywords:** children, Down syndrome, executive functions, social cognition, working memory

## Abstract

Many studies show a link between social cognition, a set of cognitive and emotional abilities applied to social situations, and executive functions in typical developing children. Children with Down syndrome (DS) show deficits both in social cognition and in some subcomponents of executive functions. However this link has barely been studied in this population. The aim of this study is to investigate the links between social cognition and executive functions among children with DS. We administered a battery of social cognition and executive function tasks (six theory of mind tasks, a test of emotion comprehension, and three executive function tasks) to a group of 30 participants with DS between 4 and 12 years of age. The same tasks were administered to a chronological-age control group and to a control group with the same linguistic development level. Results showed that apart from deficits in social cognition and executive function abilities, children with DS displayed a slight improvement with increasing chronological age and language development in those abilities. Correlational analysis suggested that working memory was the only component that remained constant in the relation patterns of the three groups of participants, being the relation patterns similar among participants with DS and the language development control group. A multiple linear regression showed that working memory explained above 50% of the variability of social cognition in DS participants and in language development control group, whereas in the chronological-age control group this component only explained 31% of the variability. These findings, and specifically the link between working memory and social cognition, are discussed on the basis of their theoretical and practical implications for children with DS. We discuss the possibility to use a working memory training to improve social cognition in this population.

## Introduction

Down syndrome (DS) is the most common genetic syndrome associated with intellectual disability (Canfield et al., 2006). So many studies have described the pattern of relative weaknesses and strengths in this population. Previous studies also suggest that social cognition and executive functions are critical abilities to ensure a better quality of life for infants, children and adults, also with DS. However, the amount of studies about the relation between social cognition and executive functions in DS is scant.

### Social Cognition in Children with Down Syndrome

The importance of competent social cognition abilities in having a satisfying personal life and social interactions is widely accepted. In this study, we understand social cognition as a set of abilities that involve cognitive capability applied to social situations (Harvey and Penn, 2010). Thus, this set of abilities includes understanding mental states and intentions in oneself and in others (or what has traditionally been known as theory of mind), emotional recognition and perception, and social knowledge, among others. According to this definition, some authors have suggested that socio cognitive abilities can be divided in two parts (Shamay-Tsoory et al., 2006; Tirapu-Ustárroz et al., 2007): a part more connected with cognitive aspects, and another part more related to affective aspects. On the one hand, from the cognitive perspective, it has been considered essential to understanding the difference between knowledge of self and others. On the other hand, from the affective perspective, the empathic appreciation of the emotional state of others has been considered essential.

This study will address both aspects of social cognition. Regarding the more cognitive aspect, we will focus on one of the most widely studied developmental milestones -the comprehension of explicit first-order false beliefs- as well as on other abilities that are acquired either before or after it. As for the more affective aspect, we will explore different dimensions of emotional comprehension above and beyond facial expression recognition.

Initially, as a result of studies like the one conducted by Baron-Cohen et al. (1985), and descriptions of people with DS as individuals who are especially friendly and interested in others, highly sociable and with few social problems, it was postulated that these children had no particular difficulty in theory of mind development.

However, subsequent research has revealed such difficulties (Binnie and Williams, 2002; Giaouri et al., 2010). For example, the study conducted by Giaouri et al. (2010) suggested that children with DS have difficulties in understanding false beliefs and appearance-reality compared with typical developing children and children with intellectual disability of unknown etiology. Molina and Amador (2010) found that when offered the necessary help children with DS are able to exhibit a similar performance to that of their peers with typical development.

With regard to the more affective aspect, most studies have focused on the recognition of facial expressions in others, this being a necessary ability to respond appropriately in situations requiring social interaction. Studies like the one by Wishart et al. (2007) show that, among all aetiologies of intellectual disability, children with DS are the only ones that exhibit a significantly lower performance than children with typical development in interpreting facial expressions. Previous studies reinforce this idea regarding difficulties with emotional recognition among children with DS (e.g., Kasari et al., 1995), some suggesting particular difficulty in recognizing fear, surprise and anger (Hippolyte et al., 2008). In fact, as suggested in the work carried out by Kasari et al. (2001), it is possible that emotional recognition among children with DS is more related to their mental than their chronological age.

### Executive Functions in Children with Down Syndrome

For a long time, there has been a lack of clarity over which abilities are included under the concept of executive functions. Some authors have posited that they are higher-order control processes, while others have defined them as processes aimed at achieving a milestone; some have emphasized the constructive and creative aspect, while others still have focused on working memory. In an attempt to encompass all these approaches, Hughes (2011) and Low and Simpson (2012) conceptualized executive functions as an umbrella term that includes a set of complex cognitive abilities that guide actions aimed at a goal and adaptive responses to new or complex situations. According with Diamond (2013), the core components of executive functions are inhibition, working memory, and cognitive flexibility. So, in our study, we will focus on them.

To succeed in life and have a healthy social, cognitive and psychological development we need to: control our attention, thoughts, emotions or behavior (called inhibitory control), hold information in mind and work with it (called working memory), and based on these two skills, change spatial and interpersonal perspectives (called cognitive flexibility). As suggested by Pennington and Bennetto (1998), people with DS are expected to exhibit deficits in these executive function skills due to the fact that they have often been described as having persistent behavior (Wilding et al., 2002). However, some studies suggest that in children with DS, it seems that difficulties with executive functions do not occur equally across all of these components (Rowe et al., 2006; Kogan et al., 2009). For example, the study by Pennington et al. (2003) only describes difficulties in the components associated with the functioning of the hippocampus (such as long-term visual and verbal memory). The one conducted by Lanfranchi et al. (2009) find them in a simultaneous task on spatial working memory but not in one on spatial sequencing. The study conducted by Carney et al. (2013) shows that compared with children with typical development of the same mental age, children and adolescents with DS have difficulty with working memory but not inhibition and fluency.

In a developmental study by Costanzo et al. (2013) designed to test the hypothesis of etiological specificity, different aspects of the executive functions were evaluated in children, adolescents and adults with DS, Williams syndrome, and a typical development group of the same mental age. Both groups with intellectual disabilities displayed difficulties with some components of the executive functions, such as selective attention and working memory, but not others, such as inhibition. A different pattern was also found according to the etiology of the disability, participants with DS displaying a more affected performance in cognitive flexibility, memory and verbal inhibition.

Recent studies have analyzed DS executive functions by parent and teacher reports like the Behavior Rating Inventory of Executive Function-Preschool (BRIEF-P; Gioia et al., 2003). On the one hand, in the study conducted by Lee et al. (2011), caregivers of children with DS completed these report. Results suggest a specific pattern of executive function weaknesses in this population; working memory was the most impaired domain and emotional control the least impaired one. On the other hand, in the study of Daunhauer et al. (2014) were the teachers of children with DS who completed the report. The results of this study suggested that in the area of school function, children with DS showed a distinct pattern of strengths and weaknesses. On the Activity Performance domain, children with DS reported greatest challenge on recreational movement, following social conventions, functional communication, positive interaction, or behavior regulation among others. On the other hand, on Task Supports domain, children with DS reported to need more assistance than adaptations, and that supports on cognitive-behavioral tasks were the subdomain in which they showed higher levels of assistance. Apart from that, it is important to highlight that executive functions was the only predictor of school function in this study. The idea of greater difficulty on the cool EF suggested by these studies was also found in the work by Lee et al. (2015).

Furthermore, some studies suggest a dissociation between verbal and visuo-spatial abilities in children with DS (Laws, 2002; Brock and Jarrold, 2005), because they show deficits in verbal but not in visuo-spatial working memory abilities (Lanfranchi et al., 2012).

So, it seems that research about executive functions indicates the presence of a particular profile of abilities and difficulties in DS children. Being some components more preserved (as inhibition or visuo-spatial working memory), and others most affected (as working memory, verbal inhibition, or cognitive flexibility). Therefore, this second group of components could require the design of interventions to improve it.

### The Role of Executive Functions in Social Cognition

In the pioneering study by Russell et al. (1991) on children with typical development, a positive association was found between performance in a false belief task and one on strategic deception task. Although deception tasks have been considered traditionally as a social cognition measure, in the study of Russell et al. (1991) has been considered as an executive control task. According with Hala and Russell (2001), children’s difficulty in this task is related with the executive control demands, particularly with the dual requirement to hold in mind the task rules and the inhibition of a prepotent response of pointing directly at the treat.

Since then, several studies have confirmed the relationship between individual differences in executive functions and individual differences in theory of mind (Carlson and Moses, 2001; Carlson et al., 2002, 2004a,b). The nature of this relationship is unclear. However, one of the perspectives argues that executive functions are needed for theory of mind (Russell, 1996, 1998; Hughes and Ensor, 2007; Austin et al., 2014). So, in this study, we will focus on the role of executive function components in social cognition.

Of all the theory of mind abilities, the most studied in this regard has been the understanding of false belief, which in general terms has been positively associated with flexibility, inhibition and working memory, but not planning.

With regard to cognitive flexibility, both correlational and training studies show the presence of a relationship between this component and theory of mind. Carlson and Moses (2001), for example, found relationships between various theory of mind tasks and a cognitive flexibility task. Specifically, Carlson and Moses (2001) found that inhibitory task requiring a novel response in the face of a conflicting prepotent response significantly predicted performance in theory of mind tasks. However, inhibitory task requiring the delay of a prepotent response was not significant in the same analysis. Additionally, Zelazo et al. (2002) found that a poor performance in theory of mind tasks might be caused by a lack of ability to integrate two contradictory rules into one system. A training study conducted by Kloo and Perner (2003) showed that false belief training improves performance in a card classification task that evaluates cognitive flexibility, and vice versa.

In relation to inhibition, Hughes (1998) found a correlation between the performance of a deception task and inhibitory control. A year later, Perner and Lang (1999) confirmed this relationship, and Carlson and Moses (2001) subsequently also found a strong association between inhibition and false belief. Further studies such as that by Carlson et al. (2002) have confirmed this association.

Regarding working memory, Olson, Kennan and colleagues (Gordon and Olson, 1998; Keenan et al., 1998) suggested that the ability to hold two conflicting perspectives on the same stimulus is a prerequisite for promoting the development of social cognition. In line with this, Davis and Pratt (1995) found that children under 4 did not succeed in false belief tasks because of difficulties in working memory. Gordon and Olson (1998) described a correlation between working memory and an appearance-reality and a false belief task. Subsequent studies have confirmed this relationship (Keenan et al., 1998; Hala et al., 2003; Mutter et al., 2006) although there are also studies that suggest the opposite (Hughes, 1998; Slade and Ruffman, 2005).

The relationship between the understanding of false belief and the executive functions has been described for different stages of development (Carlson et al., 2004a; Dumonthiel et al., 2010), in longitudinal studies (Flynn, 2006; Hughes and Ensor, 2007), when the tasks involve minimal executive demands (Perner et al., 2002; Moses and Carlson, 2004) or in populations with atypical development, such as autism spectrum disorders (Pellicano, 2007).

However, it is unclear which components of the executive functions display a stronger relationship with theory of mind abilities. Zelazo et al. (1996) examined the relationship between social cognition and executive functions in adults with DS. They found that performance in a set of theory of mind tasks and in a card sort task was positively correlated when mental age was controlled. However, we do not know more studies or results in infants or children with DS.

Therefore, the aim of this study is to take a more in-depth look at this relationship in children with DS. We want to investigate the role of executive functions in social cognition among children with DS and compare this relationship with the one described for children with typical development of the same chronological age and a similar level of linguistic development (LD). In addressing this aim we will be analyzing the social cognition and executive function abilities of children with DS in depth and will focus on how these abilities evolve with increasing chronological age and language development.

## Materials and Methods

### Participants

A total of 90 participants (aged 2;9 – 12;2) took part in the study, divided into three groups of 30 participants: one group of children with a medical diagnosis of DS, and two control groups composed of children with typical development; one with the same chronological age as the participants with DS (CA), and a second with the same level of LD. Children with other associated difficulties were excluded from the study.

As shown in **Table 1**, the CA group participants had the same chronological age and gender distribution as the DS group participants. According to the *T*-test, the age of these two groups was statistically higher than that of the LD group participants (*p* < 0.0005), while the LD group participants had a similar level of language development (±4 months) to that of the DS group participants (the gender variable was not taken into account in the construction of this control group). The *T*-test shows that the language level of the CA group participants was statistically higher than that of the DS and LD groups (*p* < 0.0005).

**Table 1:** Characteristics (sex, age, language, and IQ) of each group of participants.

			Age		Language^a^		IQ^b^	
Group	*n*	Girls (*f*)	Range	*M (SD)*	Range	*M (SD)*	Percentile ≤ 25	*M (SD)*
DS	30	18	4;0–12;2	8.54 (2.36)	1;8–8;6	4.49 (1.69)	90%	14.37 (3.45)
CA	30	18	4;0–12;2	8.54 (2.36)	3;5–14;5	8.81 (2.86)	0%	27.90 (9.61)
LD	30	16	2;9–9;1	4.52 (1.64)	2;1–8;3	4.53 (1.48)	0%	16.63 (5.19)

*^a^Language development was calculated using the score obtained in the Peabody Picture Vocabulary Test or PPVT (Dunn et al., 2006); ^b^The IQ score was obtained via administration of the Raven Progressive Matrices (Raven et al., 1996).*

### Tasks

In this paper, we evaluate two aspects of participants’ cognitive functioning: social cognition and the executive functions. Below, we detail the tasks used to evaluate each of these aspects.

#### Social Cognition Tasks

To evaluate social cognition, we used six tasks that have been traditionally used to evaluate theory of mind, and one test of emotional understanding.

We divided the theory of mind evaluation tasks into three levels of difficulty according to their distance from the understanding of first-order false beliefs, and we included two tasks in each level. Apart from that, a pilot study with DS children was conducted to ensure that participants understood the tasks (Amadó et al., 2012). We designed visual and verbal aids to compensate comprehension difficulties detected.

In Level 1, we used two tasks to evaluate abilities developed by children with typical development prior to the first-order false belief. We administered an adaptation of the task *Diverse Beliefs* designed by Wellman and Liu (2004) and an adaptation of the task *Seeing is knowing* developed by Pratt and Bryant (1990). In the *Diverse Beliefs* task children had to predict the behavior of the story character according to the beliefs of the character. It is important to know that the character always had a belief contrary to child’s. In the *Seeing is Knowing* task, we evaluated the capacity of the child to understand the relationship between seeing (or not seeing) the content of a closed box and knowing what object was inside the box. In both tasks, we used pictures to tell the story and facilitate their understanding. The score for each of these tasks was one point, meaning the highest score at this level was two points. In order to make the score at this level equivalent with the scores in other theory of mind levels, we doubled the total score for Level 1.

In Level 2, we included first-order false belief tasks. Thus, we administered the *Unexpected Content* task, based on the procedure designed by Gopnik and Astington (1988), and the *Change of Location* task, designed by Wimmer and Perner (1983). In the *Unexpected Content* task, we used a tube of Smarties^®^ with rocks inside. After exploring the tube, we showed its real content to the child. Then we asked them what they thought there was inside the tube before opening it, and what their friend would think the tube contained without seeing the real content. In the *Change of Location* task the child had to predict, in a story represented with small dolls, the behavior of a character when the character held a false belief about the location of a hidden object.

Each of the Level 2 tasks was awarded two points, meaning the highest score for this level was four points.

At Level 3, we used tasks which according to developmental research are successfully completed after the first-order false belief. Specifically, we used an adaptation of the *Deception* task designed by Sodian (1991) and a *Second-Order Change of Location* task based on the procedure devised by Sullivan et al. (1994). The *Second-order Change of Location* followed a procedure similar to that described in the first-order false belief task. However, in this story the child had to predict the behavior of a character involved in a second order recursivity situation. In the *Deception* task the child played a game, with the puppets of the Little Red Ridding Hood and the Wolf, where the player getting more stars was the winner. To win stars the child had to help Little Red Ridding Hood (who gave the stars to the chid whenever she found one) and had to deceive the Wolf (who kept the stars for himself). Each of the tasks was awarded two points, meaning the highest score for this level was four also points.

Finally, in order to evaluate emotional understanding, we administered an adaptation of the *Emotional Comprehension Test* by Albanese and Molina (2008). Of the nine components included in the original version of this instrument, we administered six, selected on the basis of results from the study by Pons et al. (2004): recognition (I), external causes (II), desires (III), beliefs (IV), memory (V), and hidden emotions (VII). In the administration of each component, we followed the instructions of the original test. Also following the instructions in the manual, each component that was passed scored one point, meaning the maximum score in this task was six points.

In all of our analyses in the results section, we consider scores from the theory of mind and emotional understanding tasks together, giving a maximum score of 18 points for social cognition.

#### Executive Function Tasks

In accordance with the results presented in the study by Miyake et al. (2000), in this study, we used different tasks to evaluate three of the components of the executive functions: working memory, inhibition, and cognitive flexibility.

To evaluate working memory, we administered a version of the visual-spatial memory task used by Lanfranchi et al. (2004), which we called the *Frog Task*. This entailed administering a total of eight tests (and two trial tests) divided into four levels of difficulty, in which the child had to follow two rules simultaneously. In this task, we used a frog and a board with squares. The frog jumped from one square to another. The child had to remember the frog starting position on the board (first rule) and to hit the table with the hand when the frog jumped into a red square (second rule). Each test was awarded one point only if the participant completed the game successfully; meaning the maximum score for the task was eight points.

To evaluate inhibition, we used a simplified version of the Stroop test, the *Day-Night Task* (Gerstadt et al., 1994). After two trial tests, we administered 16 tests in random order in which the participant had to inhibit their predominant response to a visual stimulus. We designed a Power Point presentation in which two images (a sun and a moon) appeared randomly. When the child saw the sun they had to say “night” (inhibiting the predominant response, “day”), and when the moon appeared in the screen the child had to say “day” (inhibiting the predominant response, “night”). One point was awarded for each correct answer, meaning the maximum score for the task was 16 points.

Finally, we evaluated cognitive flexibility by means of an adapted version of the *Wisconsin Card Sorting Test* developed by Fisher and Happé (2005), which comprises a card classification game using different shapes (triangle, round, square), colors (red, blue, yellow), and numbers (1, 2, 3). The experimenter put in front of the child, three stimulus cards to define the three categories where to classify the response cards. Then, the experimenter gave to the child each of the response cards and asked them to classify each card in the correct category. In accordance with the hidden classification rule governing the game in each part (color, shape, number, and color), the experimenter gave feedback to the child (correct or incorrect classification). The child had to discover the classification rules during the game. Following the recommendations of these authors, we used the number of completed dimensions (unaided) as a measure of overall success in this task. Therefore, the maximum score in this task was four points (classification criteria: color, shape, number, and color).

For a more detailed description of the procedure of the tasks used, see the appendix of the study conducted by Amadó et al. (2012).

### Procedure

We contacted the DS participants through various organizations dedicated to the care of people with this etiology of intellectual disability. Participants in the CA and LD groups were selected according to their chronological age, gender, and language development from different schools in provinces of Catalonia (Spain).

In all three groups, the parents/legal guardians were first duly informed of the purpose and requirements of the study by means of an explanatory letter requesting consent for their son/daughter’s participation. We then carried out between two and four individual sessions (at the school, foundation/association or child’s family home) in order to administer the tasks. The amount of time spent administering each of the tasks varied from one participant to another, but the order of administration was always the same: vocabulary, intelligence, executive functions (working memory, inhibition, and cognitive flexibility), and social cognition (emotional understanding, theory of mind tasks according to their order of difficulty).

In some analysis, participants with DS will be divided into different subgroups. According with their chronological age, we will distinguish between 3 subgroups with 10 participants in each group: the younger group (4;0 – 6;11), the middle group (7;0 – 9;11), and the older group (10;0 – 12;11). According with their level of language development (based on the score obtained in the PPVT by Dunn et al., 2006), we will stablish 2 groups with 15 participants in each group: low LD group (0;0 – 4;0) and high LD group (4;1 – 8;12).

## Results

As **Table 2** shows, the DS group participants scored significantly lower than the children with typical development in both control groups (CA and LD) in all of the administered tasks.

**Table 2:** Means (standard deviations) in the social cognition and executive function tasks in each group of participants.

		DS	CA	LD	Contrasts^e^	Cohen’s *d^f^*
						4.24
*Social Cognition*^a^		5.70 (3.94)	17.07 (2.05)	9.87 (5.78)	DS < CA^∗∗∗^/DS < LD^∗∗^	-0.91
						2.14

						-3.14
*Executive Functions*	*WM*^b^	2.47 (2.43)	7.20 (1.03)	4.57 (2.21)	DS < CA^∗∗∗^/DS < LD^∗∗^	-0.92
						1.85
	
						-1.94
	*INH^c^*	10.90 (5.12)	15.83 (0.46)	14.30 (2.32)	DS < CA^∗∗∗^/DS < LD^∗∗^	-1.04
						1.27
	
						-4.08
	*FLEX^d^*	1.43 (1.10)	3.90 (0.31)	2.57 (1.33)	DS < CA^∗∗∗^/DS < LD^∗∗^	-0.96
						1.89

*^a^Social Cognition (range: 0–18); ^b^ M, working memory (range: 0–8); ^c^INH, inhibition (range: 0–16); ^d^FLEX, cognitive flexibility (range: 0–4); *^e^*Contrasts were identified using the *T*-test. ^∗∗∗^*p* < 0.001; ^∗∗^*p* < 0.01; ^∗^*p* < 0.05; t.s. when *p* > 0.05; and n.s. when *p* > 1. *^f^*The values of Cohen’s *d* are presented in this order: DS-CA, DS-LD, and CA-LD.*

As **Table 2** shows, participants of the CA group obtained higher scores in all the tasks, followed by the participants of the LD group and the participants with DS, in this order.

As *T*-test shows, the mean score of participants with typical development was significantly better than the mean score of participants with DS in all the tasks. According with Cohen (1988), the effect sizes of all these comparisons are large. Also it’s important to consider that, in all the tasks, the largest effects are observed between DS and CA participants.

However, it is interesting to analyze performance in these tasks with increased chronological age and language development in DS children. On the one hand, to test the effect of chronological age we divided participants with DS into three groups: the younger group (4;0 – 6;11), the middle group (7;0 – 9;11), and the older group (10;0 – 12;11). On the other hand, to test the effect of linguistic level, we divided participants with DS into two groups based on the score obtained in the PPVT (Dunn et al., 2006): low LD group (0;0 – 4;0) and high LD group (4;1 – 8;12). The mean scores for the chronological age and linguistic level groups into which, we divided the participants with DS are shown in **Table 3**.

**Table 3:** Means (standard deviations) in the social cognition and executive function tasks in each group of participants with Down syndrome.

	*M (SD)^a^*		Social cognition^b^	Executive functions		
				WM^c^	INH^d^	FLEX^e^
*Chronological age groups*	*5.73 (0.84)*	*(A)*	2.80 (2.44)	0.70 (1.25)	9 (5.44)	0.90 (0.32)
	*8.85 (0.85)*	*(B)*	5.10 (2.28)	2.30 (1.95)	10.20 (5.81)	1 (0)
	*11.05 (0.78)*	*(C)*	9.20 (3.94)	4.40 (2.46)	13.50 (2.99)	2.40 (1.51)

*Linguistic development groups*	*3.12 (0.67)*	*(D)*	3.67 (2.64)	1.20 (1.47)	8.67 (5.47)	0.93 (0.26)
	*5.85 (1.21)*	*(E)*	7.73 (4.04)	3.37 (2.58)	13.13 (3.68)	1.93 (1.39)

*Contrasts^f^*	A-B			A < B^∗^		
	A-C		A < C^∗∗^	A < C^∗∗∗^		A < C^∗^
	B-C		B < C^∗^			B < C^∗^
	D-E		D < E^∗∗^	D < E^∗∗^	D < E^∗∗^	D < E^∗^

*^a^Means (and standard deviations) of chronological age and linguistic age (calculated using the score obtained in the Peabody Picture Vocabulary Test or PPVT by Dunn et al., 2006) of the DS groups; ^b^Social Cognition (range: 0–18); ^c^WM, working memory (range: 0–8); ^d^INH, inhibition (range: 0–16); ^e^FLEX, cognitive flexibility (range: 0–4); ^f^Contrasts were identified using the *T*-test. ^∗∗∗^*p* < 0.001; ^∗∗^*p* < 0.01; ^∗^*p* < 0.05; t.s. when *p* > 0.05; and n.s. when *p* > 1.*

As the above table shows, both chronological age and LD were relevant factors in mastering social cognition in participants with DS. Thus, as the chronological age and linguistic level of this group of children increased, their social cognition abilities improved, with significant differences observed in the group of participants with older chronological age and both linguistic level groups.

With regard to the executive functions, we saw that performance in the tasks of working memory and cognitive flexibility also improved with both chronological age and LD of participants with DS. Therefore, these two developmental factors were also relevant to mastering these two components of the executive functions. Specifically, we observed a significant improvement in working memory in the younger chronological age group and the two linguistic level groups, and a significant improvement in flexibility in the older age group and the two LD groups. In contrast, in the inhibition task, we only observed a significant improvement in the two linguistic level groups into which, we divided the participants with DS. With inhibition, it seems that chronological age was not an important factor, and in fact, the scores obtained by all three age groups for this task were very close to the maximum score.

**Table 4** below illustrates the patterns of relationship between social cognition and the executive functions for each of the groups participating in this study. To graduate the intensity of a correlation, we used the criteria described by Bisquerra (2004): *r* = 1 perfect correlation, 0.8 < *r* < 1 very high correlation, 0.6 < *r* < 0.8 high correlation, 0.4 < *r* < 0.6 moderate correlation, 0.2 < *r* < 0.4 low correlation, 0 < *r* < 0.2 very low correlation, and *r* = 0 no correlation.

**Table 4:** Correlations between social cognition and executive function components for each group of participants.

		Social cognition	Executive functions		
			*WM*	*INH*	*FLEX*
*Social cognition*		-	0.738^∗∗∗^	0.428^∗^	0.562^∗∗^
			0.581^∗∗^	0.195	-0.154
			0.800^∗∗∗^	0.319	0.669^∗∗∗^
*Executive functions*	*WM*		-	0.478^∗∗^	0.641^∗∗∗^
				0.363	-0.154
				0.268	0.791^∗∗∗^
	*INH*			-	0.289
					0.123
					0.479^∗∗∗^
	*FLEX*				-

*The values in the table are Pearson correlation coefficients (*r*) and its significance (^∗∗∗^*p* < 0.001; ^∗∗^*p* < 0.01; ^∗^*p* < 0.05; t.s. when *p* > 0.05; and n.s. when *p* > 1). The correlations appear in this order on the table: DS, CA, and LD.*

In the DS group there was a significant correlation between social cognition and all components of the executive functions evaluated. The strongest correlation was with working memory, this being both positive and high. The correlation with components of cognitive flexibility and inhibition was also positive but moderate. Similarly, in the LD group social cognition displayed a significant, positive and high correlation with both working memory and flexibility. In contrast, in the CA group a significant correlation was only found between social cognition and working memory, this being positive and moderate. It is therefore interesting to point out that working memory was the only component of the executive functions that remained constant in the relationship patterns of the three groups of participants.

It is worth noting that, as the above table shows, internal correlations between the different components of the executive functions followed different patterns in groups. The CA group is the one which displayed most divergence in relation to the other two, without significant correlations between executive function components. On the other hand, in DS and LD groups, working memory correlates with cognitive flexibility.

To evaluate the predictive capacity of each executive component, we conducted a multiple linear regression model (independent/predictive variable: score in working memory, inhibition, and cognitive flexibility; dependent variable: total score in social cognitive abilities). The results of the multiple linear regressions (using the enter method of the SPSS) pointed in the same direction, as for all three groups the regression model only included working memory as a predictive variable of score in social cognition. However, it is interesting to note that although the predictive variable of social cognition is the same for all three groups, the percentage of variance in social cognition that this component of the executive functions was able to explain was not the same. In the DS and LD groups, the model which includes only the significant predictors (constant and working memory) explained above 50% of the variability, whereas in the CA group this component of the executive functions only explained 30% of the variability (see **Table 5**).

**Table 5:** Multiple linear regression models of social cognition for each group of participants.

	Predictive variables	Standardised beta coefficients	t (*p*)	Predictive models		
				Adjusted *R*^2^	*F* (1, 28)	*p*
DS	*Constant*	–	1.334 (0.194)	0.529	33.585	0.000
	*WM^a^*	0.592	3.228 (0.003)			
	*INH^b^*	0.101	0.684 (0.500)			
	*FLEX^c^*	0.153	0.909 (0.372)			
CA	*Constant*	–	0.942 (0.355)	0.314	14.274	0.001
	*WM^a^*	0.573	3.278 (0.003)			
	*INH^b^*	-0.005	-0.030 (0.976)			
	*FLEX^c^*	-0.066	-0.401 (0.692)			
LD	*Constant*	–	-0.711 (0.484)	0.628	49.875	0.000
	*WM^a^*	0.755	3.914 (0.001)			
	*INH^b^*	0.106	0.790 (0.437)			
	*FLEX^c^*	0.021	0.099 (0.922)			

*^a^WM, working memory; ^b^INH, inhibition; ^c^FLEX, cognitive flexibility. The predictive models described in this table include only the significant predictors.*

Although this is not the aim of the present work, we have used the same procedure to analyze the predictive capacity of social cognition on each component of executive functions (note that this multiple linear regression includes only one predictive variable). As **Table 6** shows, in the DS group, social cognition was significant to predict all the components of executive functions assessed, explaining above 50% of working memoyr, 30% of cognitive flexibility, and 15% of inhibition. In a similar pattern, in the LD group, social cognition is a significant predictor for working memory (explaining above 60%) and cognitive flexibility (explaining above 40%). And finally, in the CA group, social cognition only predicts above 30% of working memory.

**Table 6:** Multiple linear regression models of working memory, inhibition, and cognitive flexibility for each group of participants.

	Dependent variables	Predictive variables	Standardised beta coefficients	t *(p)*	Predictive models		
					Adjusted *R*^2^	*F* (1, 28)	*p*
DS	*WM^a^*	*Constant*	–	-0.241 (0.811)	0.529	33.585	0.000	
		*SC^d^*	0.738	5.795 (0.000)			
	*INH^b^*	*Constant*	–	5.061 (0.000)	0.154	6.275	0.018	
		*SC^d^*	0.428	2.505 (0.018)			
	FLEX^c^	*Constant*	–	1.775 (0.087)	0.291	12.914	0.001	
		*SC^d^*	0.562	3.594 (0.001)			
CA	*WM^a^*	*Constant*	–	1.667 (0.107)	0.314	14.274	0.001
		*SC^d^*	0.581	3.778 (0.001)			
	INH^b^	*Constant*	–	21.053 (0.000)	–	–	–	
		*SC^d^*	0.195	1.050 (0.303)			
	*FLEX^c^*	*Constant*	–	8.987 (0.000)	–	–	–
		*SC^d^*	–0.154	-0.827 (0.415)			
LD	*WM^a^*	*Constant*	–	3.149 (0.004)	0.628	49.875	0.000
		*SC^d^*	0.800	7.062 (0.000)			
	*INH^b^*	*Constant*	–	15.919 (0.000)	–	–	–
		*SC^d^*	0.319	1.781 (0.086)			
	*FLEX^c^*	*Constant*	–	2.845 (0.008)	0.428	22.671	0.000
		*SC^d^*	0.669	4.761 (0.000)			

*^a^WM, working memory; ^b^INH, inhibition; ^c^FLEX, cognitive flexibility; ^d^SC, social cognition. The predictive models are described only in the components in which social cognition is a significant predictor.*

## Discussion

The aim of this study was to investigate the role of executive functions in social cognition among children with DS and compare it with that described for children with typical development of the same linguistic level and chronological age. We will first discuss the results that children with DS obtained for social cognition and the executive functions in the administered tasks, as well as their evolution by chronological age and language development. We will then discuss the role of executive functions in social cognition and comment briefly the relationship in reverse.

Previous studies have reported difficulties in social cognition among children with DS, both from the cognitive aspect (e.g., Binnie and Williams, 2002; Giaouri et al., 2010) and the emotional aspect (e.g., Kasari et al., 1995; Wishart et al., 2007). The results of our study point in the same direction, showing that participants with DS have deficits in all aspects of social cognition that, we evaluated. We would also add, in line with the findings of Kasari et al. (2001), that these difficulties, although remaining present, are not as severe when mental age (or level of LD) is taken into account.

As for mastery of the executive functions, the results of our study on children with DS were also in line with those suggested by previous research, in particular the fact that the different components of the executive functions are affected unequally (e.g., Rowe et al., 2006). In our study, participants with DS displayed less alteration in the component of inhibition, especially when language ability was taken into account. This relative preservation of inhibition compared to the other components of executive functioning has also been described in research by Carney et al. (2013) on children and adolescents with intellectual disability, and in the study by Costanzo et al. (2013) on adolescents and adults with DS and Williams syndrome. Furthermore, in the latter study participants with DS showed a greater alteration in the components of flexibility and working memory when compared with children with Williams syndrome. Danielsson et al. (2012) found that adults with intellectual disabilities have difficulties in working memory and accessing lexical items, but not in inhibition. So it would seem that this tendency continues in later stages of development.

Beyond the difficulties described in the two aspects of social cognition and the different components of the executive functions, the results of this study suggest that, at the ages studied at least, children with DS experience improvements in these abilities with increased chronological age and language development. Lee et al. (2015) found that inhibition is the only component of executive functions that improve with age in a sample of 85 youth with DS. However it is important to consider that this study assess executive functions by a report completed by parents. Molina and Amador (2010) concluded that despite the described difficulties in social cognition, when a group of children with DS were offered the necessary assistance they improved and exhibited a similar performance to that of their peers with typical development. We believe that the improvement described in participants with DS in our study supports this finding. Thus, contrary to the stagnation that has sometimes been suggested in both individuals with DS and other forms of intellectual disability, social cognition and executive function abilities improve with development, at least in the group that we have studied. However, in order to verify the presence of this improvement with increasing age in participants with DS, we would need to conduct a longitudinal study, like the one conducted by Lee et al. (2015). Our study has not a longitudinal nature. Therefore, we can only conclude that older children with DS performed better than young children with DS, because we cannot discard that older participants had better executive function abilities in early ages.

With regard to the role of executive functions in social cognition, we should first take a moment to discuss the relationship between the various components of the executive functions. Miyake et al. (2000) suggested that in the beginning of their development, executive functions may be grouped under the same domain and no differentiation is made between them. According with these authors, as development progresses, these functions can be grouped into more specialized and separated components. The results of our study could be seen to agree with this, because in participants with DS and peers of the same linguistic level, both at a lower developmental level than the control group by age, there was a high correlation between most of the executive function components evaluated. In the control group by age, however, which had a higher level of development than the above groups, the correlation between the different executive function components was non-existent. Therefore, we believe that at this age the different executive function components have become specialized, which is why the correlational analysis presented them as independent components.

Above and beyond discussion of the relationship between the different components of the executive functions, the main aim of this study was to analyze the role of executive functions in social cognition abilities in DS children.

In all three of the study groups, a relationship was found between social cognition and working memory, as described in previous studies on children with typical development (Hala et al., 2003; Mutter et al., 2006). Surprisingly, and unlike the findings of previous studies such as that by Carlson and Moses (2001) or Carlson et al. (2002), the components of cognitive flexibility and inhibition did not display any relationship with the social cognition abilities of participants in the control group by chronological age. However, a relationship was found between social cognition and cognitive flexibility in the other two groups and between social cognition and inhibition in the group of children with DS. It is worth noting that participants in the age control group obtained very close to maximum scores in all the tasks, and the lack of a relationship between these components and social cognition could therefore be caused by this ceiling effect. As the results show, participants with typical development of the age-matched control group obtained a score near the ceiling on social cognition, working memory, inhibition, and cognitive flexibility tasks. Perhaps, this ceiling effect is hiding major or subtle differences between groups. So, the results of this study need to be corroborated in future works using more appropriate instruments or tasks to evaluate social cognition and executive functions in typically developing and DS children.

With regard to the pattern of relationships between social cognition and the executive functions it’s important to refer the study conducted by Zelazo et al. (1996) in adults with DS. They found that theory of mind was correlated with cognitive flexibility. However, we have no previous studies conducted in children with DS with which to compare it at the present time. What, we can state, however, is firstly that the relationship established between social cognition and the executive functions in populations with typical development is also extended to children with DS, and secondly that the relationship pattern described in individuals with DS is similar to that displayed by their peers with typical development with similar linguistic skills.

We might therefore consider working memory, being the only component of the executive functions in our study to display a relationship with social cognition in all three groups of participants, to be an essential element in improving social cognition in children with both typical and atypical development. The nature of our study is correlational. So, we can not conclude, from our results, the presence of a causal relationship between working memory and social cognition abilities. However, our results added to the ones of other studies, point to this direction. For example, Hughes (1998) described a correlation between the understanding of false belief and working memory, the relationship remaining with age and when controlling for verbal ability (Davis and Pratt, 1995; Keenan, 1998). Though other studies, such as that by Slade and Ruffman (2005) did not find that working memory facilitated subsequent understanding of false belief in a group of children aged three to four.

Regarding the predictive capacity of working memory on theory of mind, we only know of one study that supports this hypothesis, that conducted by Davis and Pratt (1995). Said study, using a multiple linear regression analysis on children aged between three and five, showed that working memory predicts performance in a false belief task, even when controlling for age and vocabulary. Nevertheless, the authors themselves say that success in working memory is a necessary requirement for competent performance in theory of mind.

We have also explored the opposite direction of this relationship: the role of social cognition in each component of executive functions. Our results show that social cognition is a predictive variable for working memory in all the groups, and also for other executive components in children with DS and their peers with a similar LD. However, we must be cautious with these results because a recent meta-analysis conducted by Devine and Hughes (2014) found an asymmetrical pattern of relationship between social cognition and executive functions; early executive functions predict later variation in false belief understanding more strongly than vice versa.

According with Diamond (2013), we have enough empirical evidence to say that executive functions can be improved at any age across the life cycle. More specifically, training studies like that conducted by Klingberg et al. (2005) on children with attention difficulties, but also others conducted on children with typical development, suggest that the component of working memory can be trained. However, according to Shipstead et al. (2012), the aforementioned training studies displayed many shortcomings and a definitive demonstration of the possibility of improving working memory through training was therefore still required. In response to this debate, the meta-analysis conducted by Melby-Lervå and Hulme (2013) showed that working memory training produces only short-term effects and cannot be extrapolated to work with other cognitive abilities. However, in this same study the authors observed, through the analysis of a small and surely insufficient number of studies, that in visuospatial working memory the effects of training can last for up to 5 months. More recent studies report specific data about the possibility to train working memory in individuals with DS. For example, the study by Costa et al. (2015) showed that two adolescents with DS improved their performance in some (trained and non-trained) working memory tasks, specifically in visuo-spatial working memory tasks, after a six-week school-based intervention. In the same direction, Pulina et al. (2015) found that thirty-nine children and adolescents with DS improved their performance in the spatial-simultaneous component of working memory after a training administered individually (by parents or experts) during a month. It is worth noting that these improvements were maintained after a month in both groups.

Aside from the results suggested by training studies, and given that cognitive flexibility is also found to be related to working memory in children with DS and their peers of the same linguistic level, we might consider it to be another important element in improving social cognition. This was demonstrated by the findings of Fisher and Happé (2005) in research where training in the executive functions (based on cognitive flexibility) was found to be useful in improving the understanding of false belief in children with autistic disorders.

Working memory or cognitive flexibility training could be an open window for improving social cognition in this population. However, with the research available to us today, we can state that the effects of working memory training (or that of other components of the executive functions) on the understanding of false beliefs are not clear, and even less so when applied to social cognition. It is for this reason that, taking into account the contributions by Diamond (2012) regarding repeated practice as a key element in the success of executive function training and the greater benefit of this to children with poorer executive functions, we believe it necessary and useful to continue to explore this relationship in these populations.

In the future, this line of inquiry could provide the key to promote the cognitive domains of social cognition and executive functions in children with DS. But also, and more importantly, could provide the key to ensure higher levels of inclusion in society and a best quality of life for people with DS.

## Conclusion

The aim of this study was to investigate the role of the executive functions in social cognition among children with DS and compare it with that of their peers with typical development. Apart from one study of adults with DS, we do not know of any previous studies that have addressed this question in this population, and neither have, we found many studies comparing the performance of children with DS and that of their peers with typical development of the same linguistic level.

The results of our study show that, in line with the findings of previous studies, participants with DS underperformed in comparison with their peers with typical development, both in terms of social cognition and the executive functions. The most interesting finding is that the predictive role of executive funcitons in social cognition described in children with DS is similar to that exhibited by their peers with typical development with the same language skills. The results of this study confirm the importance of the different components of the executive functions in this relationship and highlight the central role of working memory. Moreover, they suggest that the executive functions may be displayed as undifferentiated in early stages of development.

## Author Contributions

All authors listed, have made substantial, direct and intellectual contribution to the work, and approved it for publication.

## Conflict of Interest Statement

The authors declare that the research was conducted in the absence of any commercial or financial relationships that could be construed as a potential conflict of interest.
